# Red cell distribution width as a surrogate marker of haemoglobinopathies in western Kenya

**DOI:** 10.4102/ajlm.v11i1.1644

**Published:** 2022-04-29

**Authors:** Benard M. Mutua, George Sowayi, Patrick Okoth

**Affiliations:** 1 Department of Medical Laboratory Sciences, School of Public Health Biomedical Sciences and Technology, Masinde Muliro University of Science and Technology, Kakamega, Kenya; 2 Department of Biological Sciences, School of Natural and Applied Sciences, Masinde Muliro University of Science and Technology, Kakamega, Kenya

**Keywords:** red cell distribution width, surrogate marker, biomarker, haemoglobinopathies, patients, western Kenya

## Abstract

**Background:**

Haemoglobinopathies are inherited haemoglobin disorders that result in anaemia characterised by erythrocyte anisopoikilocytosis. Red cell distribution width (RDW) measures anisopoikiloytosis and is readily reported by haematology analysers as a complete blood count parameter. The utility of RDW as a diagnostic marker of haemoglobinopathies in Kenya remains undetermined and undocumented.

**Objective:**

This study aimed to determine the diagnostic efficacy of RDW in discriminating haemoglobinopathy and haemoglobinopathy-free cases in Kenya.

**Methods:**

The case-control study used randomly selected haematology analyser outputs for haemoglobinopathy-free (241, 49.4%) and haemoglobinopathy cases (247, 50.1%) aged 1 month to 66 years old tested in the Aga Khan Hospital, Kisumu, and its satellite centres in western Kenya from 01 January 2015 to 31 December 2020. Results were verified using high performance liquid chromatography. The receiver operating characteristic (ROC) curve was used to evaluate the diagnostic power of RDW as a biomarker for sickle cell disease (SCD) and sickle cell trait phenotypes and β-thalassaemia.

**Results:**

The RDW showed diagnostic significance in SCD phenotypes at 21.1 ROC curve coordinate with 67.7% sensitivity, 90.0% specificity, 0.789 accuracy, 70.5% positive predictive validity, 88.8% negative predictive validity, 6.77 positive likelihood ratio, 0.36 negative likelihood ratio and 18.94 (11.4–31.4) odds ratio.

**Conclusion:**

An RDW of 21.1% is potentially a predictor of SCD haemoglobin phenotypes and should be included in the haematology screening algorithm as a critical value, above which suspected cases qualify to be investigated for SCD.

## Introduction

Haemoglobinopathies, thalassaemia syndromes, and structural haemoglobinopathy variants – haemoglobin S, haemoglobin E, haemoglobin C – are hereditary haemoglobin disorders resulting from mutations in genes encoding the haemoglobin polypeptide chain. These structural haemoglobin disorders result in functionally impaired molecules; impairment includes inefficient oxygen supply and susceptibility to destruction by the victim’s reticuloendothelial system, with consequential fatal or life-threatening severe anaemia and hypoxia.^1,2^ The World Health Organization reports that approximately 7% of the global population carry an inherited haemoglobin disorder gene and that about 300 000 infants are born with severe haemoglobin disorders annually, with over 200 000 being born in the sub-Saharan African countries.^1,3^ If proper measures are not put in place, it is estimated that between 2010 and 2050, 14 282 000 babies will be born with sickle cell disease (SCD), of which 82% will be born in sub-Saharan African countries.^4^ Haemoglobinopathies are neglected but increasing global health problems; children with SCD who live in sub-Saharan Africa have an estimated high mortality rate of 50% – 80% by age 5.^1,5,6,7,8,9,10,11^ Parents who are carriers of haemoglobinopathies have a 25% risk of genetically transferring potentially severe disorders to offspring, thus making prevention and control of these disorders difficult. Therefore, due to the recessive character of haemoglobinopathy inheritance, researchers have recommended screening for the carrier state in potentially susceptible populations.^12,13^

Studies have shown that the Kenyan population has a significant burden of haemoglobin disorders that remains undocumented as with many other sub-Saharan African countries. Non-surveillance is due to the high financial cost of haemoglobinopathy laboratory tests. These tests include the World Health Organization recommended haemoglobin electrophoresis and genotyping tests for population and newborn screening.^11,14^ Consequently, a simple model to unmask cases needs to be established.

Laboratory testing is the surest way to diagnose blood disorders; however, most affected children in African countries continue to die in early childhood, usually undiagnosed, due to the lack of effective programmes for early detection and treatment. A prospective cohort study in the Kilifi area of Kenya documented a 50% – 90% mortality rate in children under five years with SCD, which was consistent with the 50% – 80% mortality rate recorded among undiagnosed and untreated children across Africa. The authors recommended prioritising SCD diagnosis and management health research in Africa. The current study sought to determine the potency of red cell distribution width (RDW) as a surrogate marker for haemoglobinopathies circulating in a vulnerable population in resource-poor settings of western Kenya.^4,5,7,6^ The Kenyan regions served by the Aga Khan Hospital Kisumu, being within the malaria holoendemic region of western Kenya, lie in the Lake Victoria Economic Block region which is known to have a high burden of haemoglobinopathies, particularly sickle cell haemoglobinopathy.^11,15,16^ The need for a less costly haemoglobinopathies laboratory testing method in western Kenya is imperative.

Previous studies have demonstrated the utility of RDW as a discriminatory marker of iron deficiency anaemia from other microcytic anaemias while other studies have demonstrated the ability of this haematological parameter to discriminate iron deficiency anaemia and thalassaemias. The haematological index or parameter, RDW, seems able to discriminate haemoglobinopathies generally from other erythrocyte disorders associated with anaemia.^17,18,19,20,21,22^ This makes RDW a potentially simpler, cheaper, faster, and potentially dependable laboratory assay method for haemoglobinopathy detection suitable in low-income settings of western Kenya. A sickling test was recommended for screening SCD in children since it proved to have high sensitivity and specificity compared to solubility test and peripheral blood film in a study done in Uganda. However, unknown adult haemoglobinopathy carriers were left out by the same study; thus, the present study sought to unmask these carriers from the general population.^23^

The RDW measures variation in red blood cell sizes (anisocytosis) and shapes (poikilocytosis) in cell volume within the red cell population. These are erythrocyte phenotypic features that are commonly abnormal in the presence of haemoglobinopathy.^24^ The RDW is one of the haematological indices routinely generated by automated haematology analysers in clinical laboratory assays; therefore, it is a simple, faster, cheaper, and widely used test in routine practice as part of a full haemogram report. The RDW has been studied as a significant entity in various disease pathogeneses involving erythrocyte size and shape variations^24,25,26^ It is derived and presented as a coefficient of variation.

Studies have shown the potential utility of the RDW as a marker for laboratory detection of haemoglobinopathies, but there is a paucity of data on its use in Kenya. Genetic variation and environmentally and socioculturally imposed epigenetic changes have been shown to influence gene-phenotype.^27^ It is uncertain if the RDW values and their relationship with haemoglobin phenotypes on populations in other geographical settings can apply to the Kenyan scenario, especially the malaria holoendemic Lake Victoria basin. The overall goal of the study was to contribute to improving the chances of survival of infants and children with haemoglobinopathies by enabling financial access to timely laboratory testing through a dependable but affordable assay method. Its main objective was to establish the overall accuracy of the RDW as a surrogate marker of haemoglobinopathies among age-mixed patients.

## Methods

### Ethical considerations

Ethical approval was granted by the Masinde Muliro University Ethical Review Committee (reference MMU/COR:403012 vol 3(03) and National Commission of Science and Technology (NACOSTI) (ref. 407653). Permission to collect data was approved by Aga Khan Hospital, Kisumu Ethics committee (reference ADM/007/089) thus patients’ consent was not needed. The raw data was stored by the principal investigator in restricted rooms and electronic data was coded to maintain anonymity in password proof computers.

### Sample size determination

Sample size calculation was performed using Cochrans’s formula for sample size determination in case-control and other comparative studies.^28^ Assuming 19% prevalence of α-thalassaemia and sickle cell among children enrolled in a malaria vaccine clinical trial study done at Kombewa in Lake Victoria basin, western Kenya, a confidence level of 95% and a precision level of 5%, sample size of 237 was obtained; since this was a two-arm study (case-control), an equal control of 237 was needed, giving a minimum required sample size of 474.^16^

### Study design

This was a hospital-based cross-sectional retrospective comparative study of 488 randomly selected high performance liquid chromatography confirmed haemoglobinopathy, but non-iron deficiency, subjects (cases) (*n* = 247, 50.1%) and haemoglobinopathy-free and non-anaemic results (control group) (*n* = 241, 49.4%) with corresponding complete blood counts from hospital databases for the Aga Khan Hospital, Kisumu, and its western Kenya satellites.

### Data collection

Data were obtained from the laboratory database on patients examined at the hospital’s haematology laboratory for the past five years from 01 January 2015 to 31 December 2020. Complete blood count reports were performed using various Sysmex analysers (KX-21N, XP 300, SYSMEX XNL 330, SYMEX XS 500i and SYSMEX XS1000i; Sysmex Corporations, Kobe, Japan). The cases were individuals who were confirmed for various haemoglobinopathies using a high performance liquid chromatography (Bio-rad D10) machine (Bio-Rad Laboratories, Hercules, California, United States). Excluded cases included those without their respective complete blood counts reports, those with confirmed leukaemia, and those cases that had received transfusion in the past three months. The control group consisted of individuals presumed free from disorders normally associated with abnormality of red erythrocyte shape and size (including haemolytic, macrocytic or iron deficiency anaemia) and haemoglobinopathy-free. These were age-mixed people electrophoretically confirmed to have normal haemoglobin (haemoglobin AA genotype) and had haemoglobin concentrations of ≥ 9.5 g/dL for ≤ 5-year-olds, ≥ 10.5 g/dL for ≤ 12-year-olds and ≥ 11 g/dL for ≥ 13-year-olds.^29^ All participants had RDW results as part of the complete blood counts from automated haematology analysers, but cases had additional haemoglobin profiles.

### Data analysis

Statistical Package for Social Sciences version 20 (SPSS Inc., Chicago, Illinois, United States) was used to analyse data with Kolmogorov-Smirnov and Shapiro-Wilks tests. These tests revealed that the control group was a skewed (non-normal) distribution (*p* < 0.05); thus, a non-parametric statistics test, the Kruskal-Wallis H-test, was used to assess the RDW variation within haemoglobinopathy variants while the Mann Whitney U-test was used to compare the RDW variations between groups as recommended by Nahm, 2016.^30^ Accordingly, the normal RDW reference values were derived from the control group as the upper limit of 95% confidence interval (CI) of the median. Data were summarised as a median and interquartile range for the RDW and percentage for haemoglobinopathy presence. The clinical utility of the RDW was studied through receiver operating characteristic (ROC) curves analysis to assess its diagnostic efficacy in differentiating diseased (haemoglobinopathy) from non-diseased (haemoglobinopathy-free) population. The sensitivity and specificity at optimal points by use of Youden index, plus predictive values, likelihood ratio (LR), and odds ratio (OR) at the 5% significance level (*p* = 0.05) were determined.^31^

## Results

### Demographic characteristics of study participants

The proportions of the control group (49.4%, *n* = 241) and the case group (50.6%, *n* = 247) did not differ significantly (*p* = 0.740) in a total of 488 individuals (Table 1). There was no significant difference *(p* = 0.502) between the number of male (43.9%, *n* = 214) and female (56.1%, *n* = 274) participants. The majority of the participants were from the Kisumu station (49.0%, *n* = 239), followed by Busia (15.4%, *n* = 75) and Homabay (12.3%, *n* = 60; *p* < 0.001). The rest of the participants were distributed among the rest of the locations. There was a statistically significant variation in SCD frequencies across the three age groups: ≤ 5-year-olds (42.4%, *n* = 207), ≤ 12-year-olds (19.9%, *n* = 97), and ≥ 13-year-olds (37.7%, *n* = 184; *p* < 0.001).

**TABLE 1:** Demographic characteristics of study participants and RDW in control and case (haemoglobinopathies) groups in western Kenya, 01 January 2015 – 31 December 2020.

Characteristic †	Number	Percentage	Participant type								*p*
			Controls				Cases				
			*n*	%	Median	IQR	*n*	%	Median	IQR	
All participants	488	N/A	241	49.4	-	-	247	50.6	-	-	0.740
**Stations**											**< 0.001**
Busia	75	15.4	22	4.51	-	-	53	10.9	-	-	
Bungoma	42	8.6	28	5.74	-	-	14	2.9	-	-	
Kitale	17	3.5	7	1.43	-	-	10	2.1	-	-	
Kakamega	20	4.1	10	2.10	-	-	10	2.1	-	-	
Kisumu	239	49	137	28.10	-	-	102	20.9	-	-	
Kisii	24	4.9	14	2.90	-	-	10	2.1	-	-	
Homabay	60	12.3	22	4.51	-	-	38	7.8	-	-	
Migori	11	2.3	1	0.21	-	-	10	2.1	-	-	
**Gender**											
Male	214	43.9	102	20.90	-	-	112	22.9	-	-	0.502
Female	274	56.1	139	28.50	-	-	135	27.6	-	-	
**Age**											
≤ 5 years	207	42.4	82	16.80	-	-	125	25.6	-	-	**< 0.001**
≤ 12 years	97	19.9	37	7.58	-	-	60	12.3	-	-	
≥ 13 years	184	37.7	122	25.00	-	-	62	12.7	-	-	
***Haemoglobinopathies***											
**Red cell distribution width**					14.5	2.7	-	-	20.7	8.3	**< 0.001**
Haemoglobin SS disease	-	-	-	-	-	-	-	-	25.4	5.5	**< 0.001**
Homozygous SS disease with elevated Haemoglobin F	-	-	-	-	-	-	-	-	20.9	5.5	**< 0.001**
Haemoglobin SS disease combined with β-thalassaemia	-	-	-	-	-	-	-	-	23.3	7.9	**< 0.001**
SS trait (haemoglobin AS genotype)	-	-	-	-	-	-	-	-	16.4	6.5	**< 0.001**
SS trait (haemoglobin AS genotype) with elevated haemoglobin F	-	-	-	-	-	-	-	-	24.2	-	0.449
SS trait (haemoglobin AS genotype) combined with β-thalassaemia	-	-	-	-	-	-	-	-	20.9	10.5	0.791
+β-thalassaemia	-	-	-	-	-	-	-	-	19.9	8.6	1.00

Note: *P*-values in bold define a statistically significant difference; IQR could not be calculated due to small sample size for the disorder.

IQR, interquartile range; SS, sickle cell; N/A, not applicable.

† , This table shows demographic characteristics of the study participants and red cell distribution width in control and case (haemoglobinopathies) groups with their respective statistical significance.

### RDW in haemoglobinopathy phenotyping

The median RDW difference was 6.2 between the case (20.7, interquartile range [IQR] = 8.3) and the control groups (14.5, IQR = 2.7; 95%, CI = 9.1–19.9; *p* < 0.001). The RDW median for SCD phenotypes were: haemoglobin SS genotype, 25.4 (IQR = 5.5); haemoglobin SS genotype + β-thalassemia, 23.3 (IQR = 7.9); and haemoglobin SS genotype + haemoglobin F, 20.9 (IQR = 5.5) which were significantly higher compared to the control group (14.5, IQR = 2.7; 95% CI = 9.1–19.9; *p* < 0.001). Individuals with pure haemoglobin AS genotype had a significantly higher (*p* < 0.001) RDW median of 16.4 (IQR = 6.5) compared to the control group (14.5, IQR = 2.7; 95% CI = 9.1–19.9). The RDW was high in haemoglobin AS genotype + haemoglobin F (24.2, *p* = 0.449), haemoglobin AS genotype + β-thalassemia (20.9, IQR = 10.5; *p* = 0.791) and β-thalassaemia (19.9, IQR = 8.6; *p* = 1.00) patients but the difference was not statistically significant when compared to the control group (14.5, IQR = 2.7; 95% CI = 9.1–19.9).

### Diagnostic efficacy of the RDW in haemoglobinopathies

**FIGURE 1:**
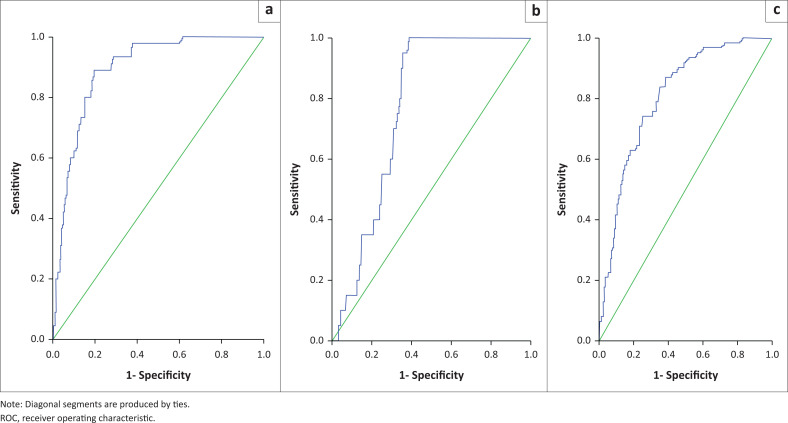
Red cell distribution width, ROC curve in SCD phenotypes (haemoglobin SS genotype, haemoglobin SS genotype + haemoglobin F, haemoglobin SS genotype + β-thalassaemia) in western Kenya, 01 January 2015 – 31 December 2020. (a) Red cell distribution width ROC curve in diagnosis of homozygous SCD. (b) Red cell distribution width ROC curve in diagnosis of sickle cell disease with haemoglobin F. (c) Red cell distribution width ROC curve in diagnosis of SCD with β-thalassaemia.

**FIGURE 2:**
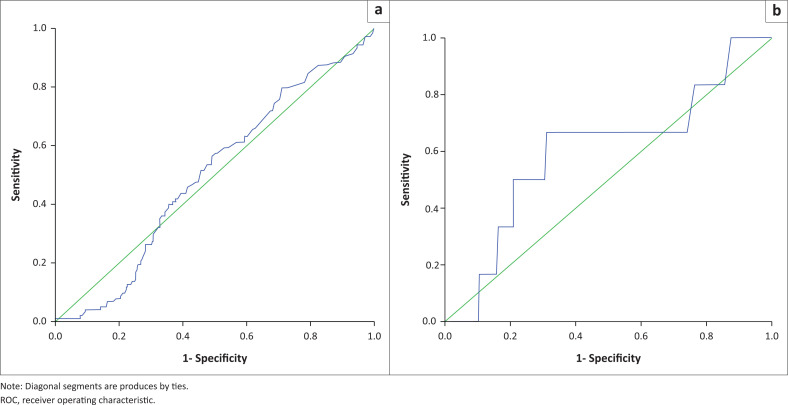
Red cell distribution width ROC curve in sickle cell triat phenotypes (haemoglobin AS genotype and β-thalassaemia) in western Kenya, 01 January 2015 – 31 December 2020. (a) Red cell distribution width, receiver operating characteristic curve for sickle cell trait flowing along diagonal line of the ROC curve. (b) Red cell distribution width, receiver operating characteristic curve for sickle cell trait+β-thalassaemia also flowing along the diagonal line of the ROC curve.

**FIGURE 3:**
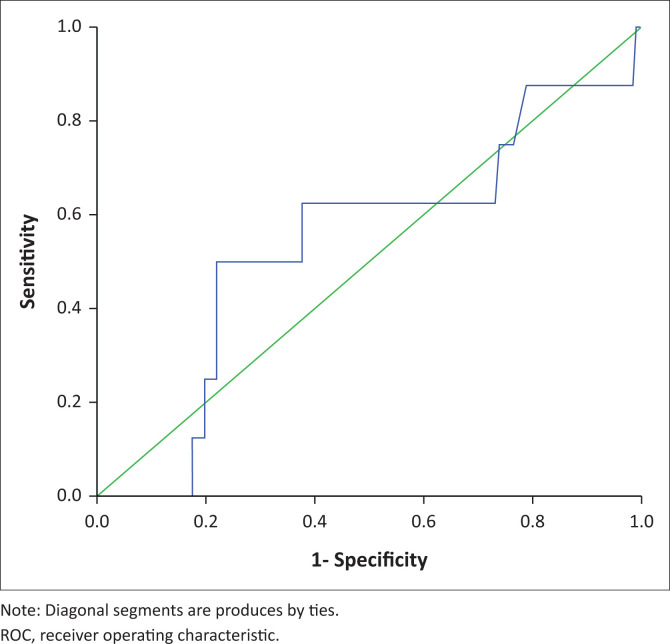
Red cell distribution width ROC curve in β-thalassaemia in western Kenya from 01 January 2015 – 31 December 2020.

At given optimal points, the RDW demonstrated its diagnostic efficacy by marking some haemoglobinopathies with a high sensitivity, specifity, Youden index and asymptotic significance (*p* < 0.001) with their ROC curves flowing upwards on the left side of the curve (Table 2; Figure 1). On the other hand, the diagnostic utility of RDW for some haemoglobinopathies was marked by low sensitivity, specifity and Youden index and did not have asymptotic significance with their ROC curve flowing along the diagonal line (Figure 2 and Figure 3).

**TABLE 2:** Summary of RDW predictive ability in haemoglobin disorders in western Kenya, 01 January 2015 – 31 December 2020.

Haemoglobinopathies	*n*	Area under curve /Youden index/Accuracy	Asymptotic Significance (*p*)	Optimal point (%)	Sensitivity (%)	Specificity (%)
Sickle cell disease	45	0.892	< 0.001	21.1	86.7	80.0
Sickle cell disease with haemoglobin F	20	0.766	< 0.001	20.8	70.0	67.6
Sickle cell disease with β-thalassaemia	62	0.805	< 0.001	17.7	78.0	64.5
Sickle cell trait	103	0.501	0.976	15.1	61.2	39.5
Sickle cell trait with haemoglobin F	2	N/A	N/A	N/A	N/A	N/A
Sickle cell trait with β-thalassaemia	6	0.600	0.399	19.8	50.0	70.0
β-thalassaemia	9	0.539	0.706	16.8	63.0	60.0
Total	247					
Haemoglobin SS phenotypes receiver operating characteristic curve.†	-	0.789	< 0.001	21.1	67.7	90.0

Note: Positive predictive value = 70.5%; Negative predictive value = 88.8%; Positive likelihood ratio = 6.77; Negative likelihood ratio = 0.36; Odds ratio = 18.94.

SS, sickle cell; N/A, not applicable.

† , This table gives a summary of red cell distribution width predictive ability in terms of Youden index/Accuracy (area under the curve), asymptotic significance (*p*), sensitivity (%) and specifity (%) at given optimal points in ROC curves. The RDW had diagnostic (asymptotic) significance in sickle cell disease (haemoglobin SS genotype) phenotypes; thus its predictive values, likelihood and OR was studied at 21.1% optimal point as shown on the table.

### Red cell distribution ROC curve in SCT phenotyping

The RDW proportion for pure haemoglobin AS genotype was 21.1% (*n* = 103; ROC curve flowed along the diagonal line with a Youden index = 0.501; *p* = 0.976) for diagnosis of pure sickle cell trait (SCT) phenotype (Table 2, Figure 2). Similarly, the RDW ROC curve coordinates at the optimal point of 19.8 did not have diagnostic significance (*p* = 0.399, sensitivity, 50%, specificity 70%; curve flowed along the diagonal line with a low Youden index = 0.600) for the diagnosis of haemoglobin AS genotype + β-thalassaemia.

### Red cell distribution ROC curve in SCD phenotyping

The RDW proportion for haemoglobin SS genotype was 9.2% (*n* = 45; optimal point = 21.1; *p <* 0.001; accuracy = 0.892; sensitivity = 86.7%; specificity = 80.0%) (Table 2, Figure 1) pushing the curve to the left upper side of the ROC curve. The proportion for haemoglobin SS genotype and haemoglobin F was 4.1% (*n* = 20; optimal point = 21.8; *p <* 0.001; sensitivity = 70.0%; specificity = 67.6%; Youden index = 0.766), with the ROC curve flowing above the diagonal line. A haemoglobin SS genotype + β-thalassaemia prevalence of 12.7% (*n* = 62) was obtained and at 17.7 the RDW optimal point (*p <* 0.001; sensitivity = 78.0%; specifity = 64.5%; Youden index = 0.805).

### ROC curve in β-thalassaemia

The RDW optimal point of 16.8 for β-thalassaemia was not significant (*p* = 0.706). The ROC curve flowed along the diagonal line area under curve = 0.539 with sensitivity = 63% and specificity = 60%) (Table 2; Figure 3).

### Sensitivity and specificity of RDW in SCD phenotype diagnosis

**FIGURE 4:**
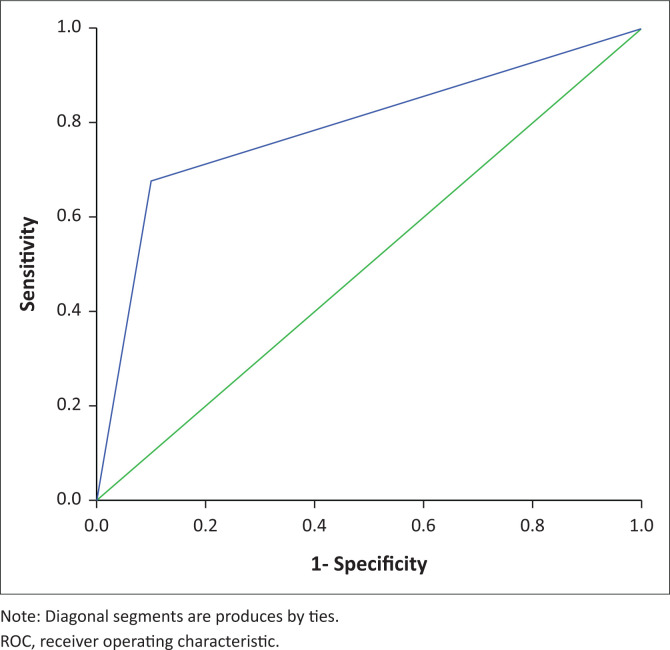
Red cell distribution width ROC curve in haemoglobin SS phenotypes in western Kenya, 01 Janauary 2015 – 31 December 2020. This ROC curve demonstrates the efficacy of RDW at 21.1% optimal point in SCD (Haemoglobin SS) phenotypes diagnosis.

The ROC curves grouped haemoglobinopathies into two groups serving as an excellent significant (*p* < 0.001) biomarker in SCD phenotypes diagnosis; but it was poor in the diagnosis of SCT phenotypes and β-thalassemia (low Youden index, sensitivity, and specificity) (Table 2). Therefore, the efficacy of RDW as a biomarker for use in SCD phenotype diagnosis was evaluated in a single ROC curve (Figure 4) giving a sensitivity of 67.7%, specificity of 90.0% and an accuracy of 0.789 at an optimal point of 21.1 (Table 2).

#### Predictive validity, likelihood and OR of RDW in SCD phenotyping

The RDW at 21.1 optimal value, recorded 70.5% positive predictive validity and 88.8% negative predictive validity in SCD phenotypes diagnosis (Table 2). Similarly, the same optimal value had a 6.77 positive likelihood ratio (LR+), 0.36 negative likelihood ratio (LR-) and 18.94 OR in the diagnosis of haemoglobin SS phenotypes.

## Discussion

The RDW was able to diagnose SCD phenotypes significantly (*p* < 0.001), but could not diagnose SCT phenotypes and β-thalassaemia (*p >* 0.05; low Youden index, sensitivity, and specificity). The overall accuracy of the RDW proved to be an excellent biomarker for SCD haemoglobinoathies; thus, unknown (seemingly haemoglobinopathy-free) cases with RDW above 21.1 need to be confirmed using advanced technology. Therefore, countries with limited financial resources who have not implemented newborn and population screening can use this potential biomarker as a cost-effective approach.

A worthless test has a Youden index of 0.5, poor sensitivity of 50%, a specificity of about 50% and a ROC curve that flows along the diagonal line and thus is unable to distinguish diseased from non-diseased individuals.^31^ The RDW could not serve as a biomarker for pure SCT (*p* = 0.976) and SCT+β-thalassaemia (*p* = 0.399) diagnoses; both had low Youden index, sensitivity and specifity.

In homozygous SCD, the RDW had a sensitivity of 86.7%, specificity of 80% and an accuracy of 0.892 which are features of a significant (*p <* 0.001) biomarker at 21.1 ROC curve coordinate.^31^ Similar findings of elevated RDW were documented by Webster and Castro^32^ with homozygous SCD having the highest RDW, followed by heterozygous SCD+β-thalassaemia and then SCD and haemoglobin F. The RDW proved to be an excellent diagnostic marker for SCD and haemoglobin F at 20.8 ROC curve coordinate where a sensitivity of 70.0% and a specificity of 67.6% were obtained with a high accuracy of 0.766.

The ROC curve demonstrated that the RDW was an excellent diagnostic marker (*p <* 0.001) in detecting SCD+β-thalassaemia with an accuracy of 0.805. The β-thalassaemia ROC curve flowed along the diagonal line with poor diagnostic significance (*p* = 0.706) similar to Qurtom et al’s report^33^ of β-thalassaemia having a normal or mildly elevated RDW at a mean of 15.4 ± 1.21, meaning it is not possible to use the RDW to differentiate β-thalassemia from the healthy population.

The RDW ROC curves were able to discriminate (*p <* 0.001) SCD phenotypes from the healthy population but could not diagnose SCT phenotypes and β-thalassaemia. The optimal RDW value for the overall efficacy in diagnosing SCD phenotypes was 21.1 (sensitivity = 67.7%; specificity = 90.0%; accuracy = 0.789). This means that in a population, an RDW > 21.1 can identify 67.7% of the individuals as having SCD while an RDW of < 21.1 will identify 90% of individuals who would test negative for SCD. This implies that at 21.1, the RDW can be used as an optimal diagnostic biomarker for SCD phenotypes in western Kenya.

A positive predictive value tells how likely an individual will test positive for a given disease. In regard to the present study, patients with RDW > 21.1 were 70.5% likely to test positive for SCD indicating 29.5% would still test negative for SCD even when the RDW was > 21.1. Negative predictive value tells how likely an individual will be to test negative for a particular disease meaning 88.1% of patients with RDW < 21.1 would be free from SCD while 11.9% would still test positive. Therefore, the RDW is proving to be a good diagnostic biomarker for SCD phenotypes among haemoglobinopathies listed in the present study.

To this end, this is the first-ever attempt to determine the likelihood of using the RDW value to diagnose SCD phenotype in western Kenya. Likelihood ratios are clinically more useful than sensitivity and specificity in determining the usefulness of diagnostic tests. The positive likelihood ratio (LR+) expresses how likely a test is going to correctly diagnose the presence of the condition where the greater the LR+, the more likely the test is going to give a true positive diagnosis. The RDW > 21.1 had a LR+ of 6.77, meaning any individual having the RDW > 21.1 is 6.77 times more likely to test positive for SCD. Negative likelihood ratio (LR–) is defined by Akobeng^34^ as the ratio of how likely a test will correctly diagnose the absence of a condition whose value is usually < 1; the closer the value gets to zero, the better the test is correctly indicating the absence of the condition. Regarding the present study, LR– was 0.36, meaning the probability of a person with the RDW < 21.1, is 0.36 times less likely to be free from SCD. It is important to note a test having both LR+ and LR– close to 1 has little influence to predict the presence or absence of a disease and is, therefore, worthless in clinical practice. On the same optimal value, an OR of 18.9 was obtained meaning that individuals with the RDW > 21.1 were 18.9 times at greater risk of having SCD haemoglobinopathy compared to those with the RDW < 21.1.

### Limitations

Sickle cell traits +haemoglobin F and +β-thalassaemia recorded abnormally elevated RDW that did not have statistical significance due to the small sample size.

### Conclusion

These findings indicate that the RDW is a promising diagnostic marker for SCD phenotypes; thus, RDW of 21.1 should be included in the haematology policy screening algorithm as a critical value above which the unknown cases qualify to be investigated for sickle cell haemoglobinopathy. However, the data used were retrospective and hence the diagnostic utility of this haematological index for haemoglobinopathy should be explored further using prospective data. A confirmatory test would still be needed and therefore provision and use of powered mini-electrophoretic equipment will be appropriate in low-resource settings.
